# Determining spatial units for modeling regional nonnative invasive plant species spread in the southern US forestlands: using the state of Alabama as an example

**DOI:** 10.48130/forres-0024-0010

**Published:** 2024-04-11

**Authors:** Sunil Nepal, Martin A. Spetich, Zhaofei Fan

**Affiliations:** 1 College of Forestry, Wildlife and Environment, Auburn University, 602 Duncan Drive, Auburn, AL 36849, USA; 2 USDA Forest Service Southern Research Station, 100 Reserve Street, Hot Springs, AR 71902, USA

**Keywords:** Nonnative invasive plant species (NNIPS), Invasion severity, Multiscale modeling framework, FIA, Spatial lag model

## Abstract

Nonnative invasive plant species (NNIPS) cause significant damage to the native forest ecosystems in the southern United States forestlands, such as habitat degradation, ecological instability, and biodiversity loss. Taking the state of Alabama as an example, we used more than 5,000 permanent United States Department of Agriculture-Forest Service's Forest Inventory and Analysis (FIA) plots measured between 2001 and 2019 over three measurement cycles to test the suitable modeling unit for quantifying invasion patterns and associated factors for regional NNIPS monitoring and management. NNIPS heavily infest Alabama's forestlands, and forestlands plagued with at least one NNIPS have increased over time: 41.1%, 50.8%, and 54.8% during the past three measurements. *Lonicera japonica* (Thunb.) was the most abundant NNIPS in Alabama, with at least 26% of its forested lands infested. The FIA data were aggregated with multiple spatial units: five levels of hydrological units, three levels of ecological units, and a county level. Invasion indices were calculated for all spatial units based on NNIPS' presence/absence and average cover in each plot. The best modeling unit was identified based on Moran's test, with the county-level modeling unit providing the best Moran's I value over all measurement periods. Influencing factors of invasion were evaluated based on spatial lag models. Our models show that the invasion index decreased with increases in public forest areas in a county. In contrast, the human population density of neighboring counties positively influenced the invasion index.

## Introduction

Nonnative invasive plant species (NNIPS) significantly impact the US economy and native ecosystems^[1]^. Despite control efforts, invasions have increased noticeably throughout the landscape. Forest ecosystems are vulnerable to invasions because invasive species can quickly alter species composition and ecosystem structure and functionality, causing a loss of forest productivity and diversity^[2]^. Southern US forest ecosystems are experiencing increasing threats from NNIPS, which displace native species, degrade fundamental forest structure and functionality^[3,4]^, and damage the environment and local economies^[5,6]^. Moreover, invasive species are expected to increase in geographic range over time, causing large-scale ecological instability of native forests and making control and mitigation measures more costly and challenging^[7]^.

NNIPS are increasing at an alarming rate throughout southern US forests; however, only partial monitoring and a few invasive control practices are being implemented^[8, 9]^. Miller^[9]^ documented 33 NNIPS rapidly growing in the southern US. Among them, some common NNIPS are Japanese honeysuckle (*Lonicera japonica* Thunb), kudzu (*Pueraria montana* (Lour.) Merr.), privet (*Ligustrum* L.), Tree-of-Heaven (*Ailanthus altissima* (Mill.) SwingleCh), silk-tree (*Albizia julibrissin* Durazz.), and Chinese tallow tree (*Triadica sebifera* (L.) Small), primarily introduced into the United States as ornamental or for multipurpose^[9−11]^. These species are more vigorous in the introduced habitat than in the native habitat^[12, 13]^, tolerant to multiple adverse conditions, have various means for seed dispersal and propagation, and grow more rapidly than most native species^[9−11]^. Two driving factors make them more vigorous in introduced areas: advantageous competitive capacity and lack of natural enemies in newly introduced areas^[14]^. Further, disturbed habitats, such as ecosystem edges, including transportation networks, are susceptible to invasion^[15]^.

Alabama is one of the most densely forested states in the southern US, with more than 68% covered with diverse and highly productive forests^[16]^. As such, Alabama depends immensely on its forests for its economy and the well-being of its residents^[17]^. As elsewhere in the southern US, Alabama's forests are experiencing increasing threats due to the invasion of NNIPS. The Alabama Invasive Plant Council has identified these seven NNIPS as extended and dense infested species in the state's managed forested lands: Chinese tallow tree, privet, Japanese honeysuckle, Japanese climbing fern, kudzu, cogongrass (*Imperata cylindrica* (L.) Beauv.), and Nepalese browntop (*Microstegium vimineum* (Trin.) A. Camus)^[18]^. These invasive species have plagued vast forestlands and become a severe problem for forest landowners in Alabama.

The invasion and spread of NNIPS in forestlands are driven by multiple factors interacting across different spatial and temporal scales^[19]^. Accordingly, their distribution is non-stationary and varies in space and across ecosystems. In addition to climatic and geographic factors (e.g., temperature, precipitation, site productivity, forest types), socioeconomic factors such as ownership, land-use change, road density, and resource management intensity and history have also been shown to be significant determinants of invasive incidence^[5, 20]^. It has often been observed that native and invasive species abundance follows a negative relationship at fine scales but roughly a positive relationship at broad scales (e.g., a forest landscape/watershed)^[21]^.

Selection of appropriate spatial scales (units) is needed for regional NNIPS modeling and monitoring. Appropriate spatial scales or units should not only help characterize the distribution patterns of NNIPS but also provide a convenient framework for monitoring and management efforts. For this purpose, we chose a group of hydrological, ecological, and administrative or political units to test their feasibility. Hydrologic units are spatially homogeneous in mass movement and energy exchange. They are better determinants of significant hydrological, ecological, and socioeconomic processes influencing the invasion and spread of invasive species^[22, 23]^. Ecological units such as provinces, sections, and subsections are generally defined based on climate, vegetation, terrain, and elevation, reflecting ecological assemblies' spatial heterogeneity and hierarchy^[24]^. In contrast, counties are mainly political and administrative boundaries for policy making and management activities primarily related to human well-being. Our primary goal was to identify the best modeling units and develop the geospatial model to examine invasion severity in Alabama. Specifically, this study aimed to 1) identify the best hierarchical geospatial modeling unit to map the extent and spread of NNIPS and 2) quantify associated factors that significantly affect the invasion and spread of NNIPS in Alabama's forestlands. These analyses provide baseline information on invasive species modeling and suitable management units for developing better prevention and management strategies to control or mitigate the negative impact of invasive species on Alabama's forestlands.

## Materials and methods

### Forest inventory and analysis (FIA) data

We obtained data from more than 5,000 FIA plots/subplots for the state of Alabama (FIA DataMart 2019). The FIA data were downloaded from the USDA Forest Service's Forest Inventory and Analysis (FIA) DataMart (Forest Inventory and Analysis Program 2018). Alabama has 5,657 FIA plots, approximately 4.9-km × 4.9-km spacing throughout the state, that is, one plot for roughly every 24.3 km^2^, to collect forest information^[16, 25]^. We used plots measured three times between 2001 and 2019 on accessible forested lands. Each FIA plot has four nested subplots; there were more than 22,000 subplots in each measurement throughout the state. Some of these were inaccessible, and some were not remeasured; as such, we found that 15,240 subplots were accessible and remeasured three times. Invasive species information such as presence and cover percent were obtained from the 'AL_INVASIVE_SUBPLOT_SPP' table, and plot level information was obtained from the 'AL_PLOT' and 'AL_COND' tables. Publicly available FIA data provide the approximate latitude and longitude due to a privacy provision. Most annual plots were within +/− half a mile of the approximate locations, and some plots were swapped^[26]^. Thus, we obtained actual FIA plot locations from the USDA Forest Service and used them for this analysis.

### Land use/forest types/demographic data in Alabama

**Figure 1:**
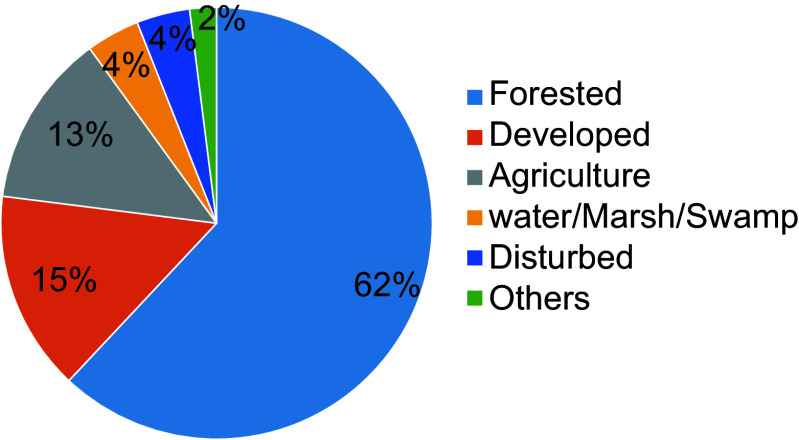
Land-use in Alabama based on LANDFIRE data (SAF/SRM classes in 2016).

We used the LANDFIRE (Landscape Fire and Resource Management Planning Tools) dataset for this analysis. The 2016 existing vegetation type (EVT) data were downloaded from https://landfire.gov/viewer/viewer.html. The EVT provides information about the existing distribution of plant communities^[27]^. Data come in 30 m × 30 m pixels and are available for the conterminous US. We used SAF_SRM classes, the crosswalk to Society of American Foresters (SAF), and the Society for Range Management (SRM) cover types. For the state of Alabama, LANDFIRE classified 48 SAF/SRM classes. We reclassified these into six major categories (Fig. 1).

Forest ownership per modeling unit was generated using FIA data. Additionally, the USDA Forest Service's forest type raster imagery (https://data.fs.usda.gov/geodata/rastergateway/forest_type/index.php), which was based on 2002 and 2003 inventory and prepared by Ruefenacht et al.^[28]^. The geoprocessing was done in ArcGIS Pro (version 2.5.0); the identity tool was used to overlap modeling units with source data (i.e., land-use and forest types) and the area on each land-use class and forest type within the modeling unit was obtained. The cover percent by each land-use class and forest type was calculated and used as an independent variable in the spatial lag model. Demographic data including human population density and the number of households per modeling unit were prepared using 2016 US Census data. Further, road density and length per unit were made based on the interstate, US Highway, and state Highway information available on the ESRI website (www.arcgis.com/home/item.html?id=fc870766a3994111bce4a083413988e4). Details of these variables can be found in Table 1.

**Table 1:** Variables used in the spatial lag model to evaluate the potential driving factors of NNIPS invasions.

Variable	Variable definition	Data types	Data description and source
public_own_pct	Percent of publicly owned forest (0−100)	Ownership	FIA DataMart (https://apps.fs.usda.gov/fia/datamart/CSV/datamart_csv.html)
rd_length	Total length of major roads (interstate and state highways) (m)	Roads	Esri (www.arcgis.com/home/item.html?id=fc870766a3994111bce4a083413988e4)
rd_density	Road density in each county (m/m^2^)		
elm_ash_cot	Elm/Ash/Cottonwood group area in percent (0−100)	Forest groups	USDA Forest Service (forest types/groups are based 2002 and 2003 data) https://data.fs.usda.gov/geodata/rastergateway/forest_type/index.php
lob_short	Loblolly/Shortleaf Pine group area in percent (0−100)		
long_slash	Longleaf/Slash Pine group area in percent (0−100)		
oak_gum_cypress	Oak/Gum/Cypress group area in percent (0−100)		
oak_hickory	Oak/Hickory group area in percent (0−100)		
oak_pine	Oak/Pine group area in percent (0−100)		
lob	Loblolly Pine area in percent (0−100)	Forest types	
lob_hard	Loblolly Pine/Hardwood area in percent (0−100)		
long	Longleaf Pine area in percent (0−100)		
mix_hard	Mixed Upland Hardwoods area in percent (0−100)		
sw_no_wo	Sweetgum/Nuttall Oak/Willow Oak area in percent (0−100)		
wo_ro_hi	White Oak/Red Oak/Hickory area in percent (0−100)		
pop_2010	Population in 2010	Demographics	2010 US Census demographic information. Downloaded from Esri (https://hub.arcgis.com/datasets/esri::usa-counties/about)
pop_den_2010	Population density in 2010 (population·m^2^)		
households	Number of households in 2010		
pop_2010_nbh	Avg. population of neighborhood counties		
pop_den_2010_ nbg	Avg. population density of neighborhood counties (population·m^2^)		
ag_pct	Agriculture lands in percent (0−100)	Land-use	Land-use in 2016 from downloaded from LandFire (https://landfire.gov/viewer/viewer.html)
dev_pct	Developed lands in percent (0−100)		
dist_pct	Disturbed lands in percent (0−100)		
fr_pct	Forest lands in percent (0−100)		
ot_pct	Other lands in percent (0−100)		
wa_pct	Water cover in percent (0−100)		
area	Total county area (m^2^)		

### Multiscale modeling units

**Figure 2:**
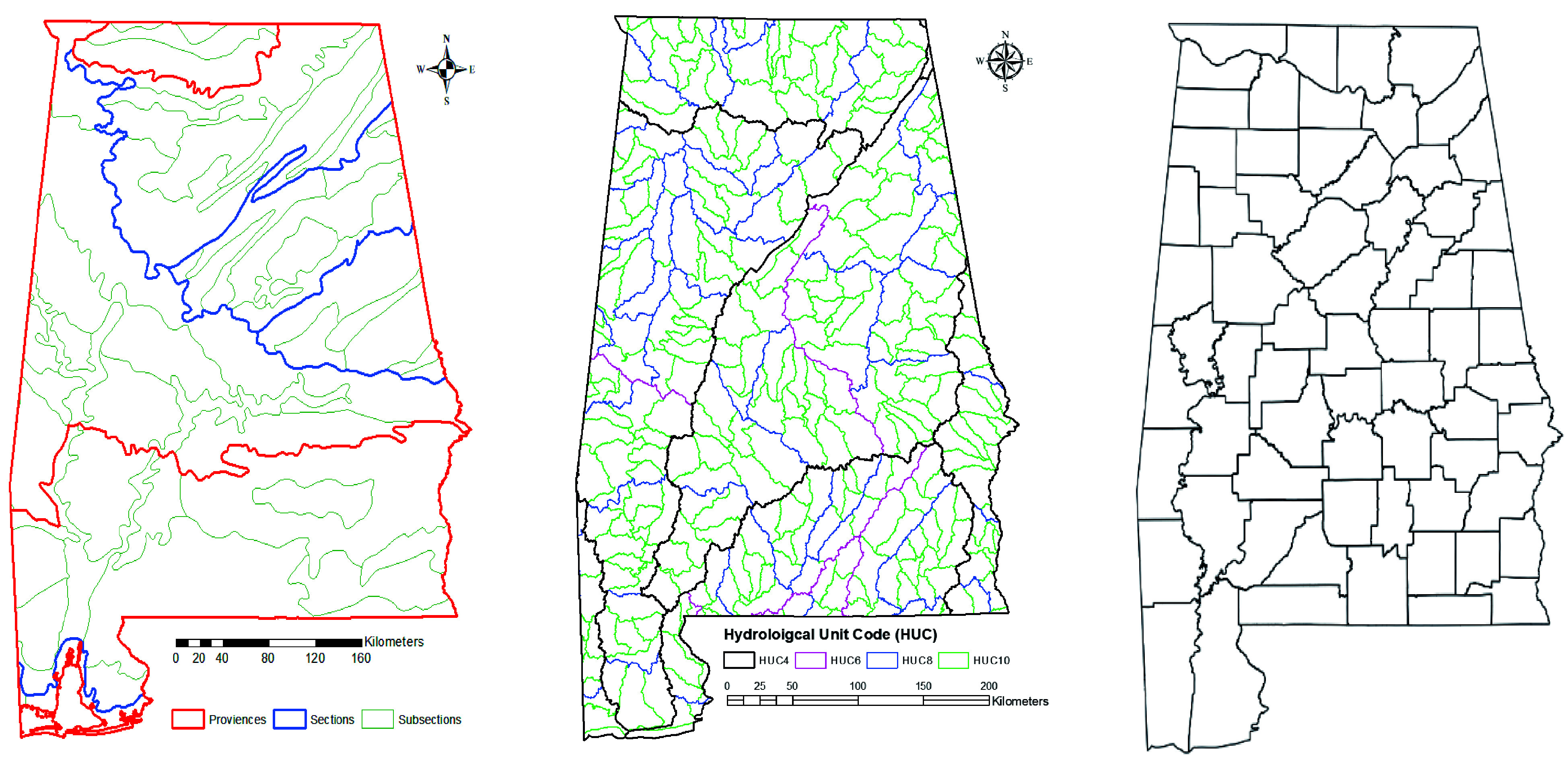
Maps of spatial units (from coarse to fine): three levels of ecoregions (left), four levels of hydrological units (middle), and counties (right) used to predict the invasion of NNIPS in Alabama's forestlands.

The FIA data were aggregated into multiple spatial units: five levels of hydrological units (HUC4, HUC6, HUC8, HUC10, and HUC12), three levels of ecological units (province, section, and subsection), and a county level. Watershed Boundary Dataset (WBD) was downloaded from the USDA Natural Resources Conservation Service (https://datagateway.nrcs.usda.gov/) (Fig. 2). The shapefiles of three-level ecological units were obtained from the USDA Forest Service website (https://data.fs.usda.gov/geodata/edw/datasets.php).

### Data preparation

Based on FIA data, we identified seven top NNIPS in Alabama: Japanese Honeysuckle, Privet, Japanese climbing fern, Sericea Lespedeza, silk tree, Chinese tallow tree, and Rose (*Rosa* L.). These species were found at least in 200 subplots (i.e., > 1.3% of all subplots) measured between 2013 and 2019. A binary variable was created to represent the presence or absence of invasive species from each subplot. To capture temporal changes, we traced the 15,240 subplots remeasured three times. Alabama's FIA plots measured between 2001 and 2005 (i.e., in cycle 8) were treated as first-time measurement (i.e., T1), 2006−2012 (i.e., cycle 9) as second-time measurement (i.e., T2), and 2013−2019 (i.e., cycle 10) as third-time measurement (i.e., T3). The number of infested subplots by each species and per measurement time is summarized in Table 2. For the NNIPS above, their presence probability (Eqn 1), average cover percent (Eqn 2), and invasion index (Eqn 3) in forestland were calculated for each modeling unit.

**Table 2:** Changes in the infestation rate (%) of NNIPS over time in Alabama's forestlands.

Measurement cycle	Year	Total subplots	Infested subplots	Infestation %	Invasive species count	Average species count per subplot (in infested subplots)
T1	2001−2005	15,240	6,268	41.1	8,251	1.32
T2	2006−2012	15,240	7,744	50.8	11,405	1.47
T3	2013−2019	15,240	8,347	54.8	14,020	1.68

1 $ {\mathrm{P}}_{\mathrm{i}\mathrm{j}}=\dfrac{{\mathrm{S}}_{\mathrm{i}\mathrm{j}}}{{\mathrm{N}}_{\mathrm{i}\mathrm{j}}} $

Here, $ {\mathrm{P}}_{\mathrm{i}\mathrm{j}} $ is the presence probability of NNIPS in forestland for a modeling unit *i* at measurement *j*. $ {\mathrm{S}}_{\mathrm{i}\mathrm{j}} $ is the number of infested subplots in a modeling unit *i* and measurement time *j*, and $ {\mathrm{N}}_{\mathrm{i}\mathrm{j}} $ is the total number of subplots in a modeling unit *i* at measurement time *j*. Here, measurement time *j* = T1, T2, and T3, and modeling unit *i* = Hydrological units (HUC4, HUC6, HUC8, HUC10, and HUC12), county, and ecological units (province, section, and subsection).

2 $ {\mathrm{C}}_{\mathrm{i}\mathrm{j}}=\dfrac{{\sum \mathrm{c}}_{\mathrm{i}\mathrm{j}}}{{\mathrm{N}}_{\mathrm{i}\mathrm{j}}}$

Here, $ {\mathrm{C}}_{\mathrm{i}\mathrm{j}} $ is the average cover of NNIPS in forestland for a modeling unit *i* at measurement time *j*. $ {\sum \mathrm{c}}_{\mathrm{i}\mathrm{j}} $ is sum of cover percent of all invasive species from all subplots found in a modeling unit *i* at measurement time *j,* and $ {\mathrm{N}}_{\mathrm{i}\mathrm{j}} $ is the total number of subplots in a modeling unit *i* at measurement *j*. The invasion index, measuring the invasion severity, was calculated as,

3 $ \mathrm{I}\mathrm{n}\mathrm{v}\mathrm{a}\mathrm{s}\mathrm{i}\mathrm{o}\mathrm{n}\;\mathrm{i}\mathrm{n}\mathrm{d}\mathrm{e}\mathrm{x}={\mathrm{P}}_{\mathrm{i}\mathrm{j}}\times {\mathrm{C}}_{\mathrm{i}\mathrm{j}} $

If any polygons in the selected modeling units had missing values, those missing values were adjusted with imputed values based on the Inverse Distance Weighted (IDW) imputation method. Addressing missing values is critical because some modeling units are small- as such, no FIA plots fall under them, resulting in NULL or missing values.

### Data analysis

Spatial and temporal trends of NNIPS were examined and graphically illustrated. Invasive species' occurrence and severity often follow clustered patterns^[29, 30]^. Moran's I test statistic, proposed by Moran^[31]^, can be used to quantify the crowdedness. A positive Moran's I statistic suggests clustering, meaning the data have a positive spatial autocorrelation^[32]^. In this analysis, we chose the modeling unit with the highest Moran's I statistic, suggesting a wider spatial variation in the data. In such a case, we need a geospatial model to account for those spatial autocorrelations. Thus, influencing factors for invasion indices were modeled using the spatially lag model- spatial autoregressive (SAR) (Eqn 4). The SAR model assumes a lag effect on the dependent variable (i.e., invasion index) by neighbors. For example, a county's invasion index is affected by nearby counties' invasion indices.

4 $ \mathrm{Y}=\mathrm{\rho }\mathrm{W}\mathrm{Y}+\mathrm{X}\mathrm{\beta }+\mathrm{\varepsilon } $

Here, **Y** is the dependent variable (invasion index), **X** represents independent variables (land-use, forest types, road density, population density, and other variables in Table 1), ρ is a parameter of spatial lag coefficient, **W** is the spatial weight matrix, *β* is the regression coefficients to be estimated, and **ε** is residuals.

Geoprocessing, data preparation, and visualization were done in Esri ArcGIS Pro 2.5.0 software and R^[33]^. Moran's I and spatial lag tests were conducted using the 'spdep' package^[34]^ in R. Highly correlated (correlation coefficient > 0.6) variables were removed from the model. The best-fitted spatial lag model was identified by choosing the lowest Akaike information criterion (AIC).

## Results

### Invasive species increased over time

The distinct species count of NNIPS in Alabama's forestlands tended to increase over time. In total, 25, 26, and 33 unique NNIPS were recorded in T1, T2, and T3, respectively. Infestation % tended to increase over time as well; during the first measurement, only 41.1% of total remeasured subplots were infested, but during the third measurement, the infestation rate had risen to 54.8% (Table 2). Alabama forestlands are not only increasing in infestation % but also adding more different invasive species over time. For instance, in T1, the infested subplots had an average of 1.32 unique NNIPS. However, in T3, 27% more (i.e., 1.68 unique nonnative invasive species) were found in Alabama forestlands.

**Figure 3:**
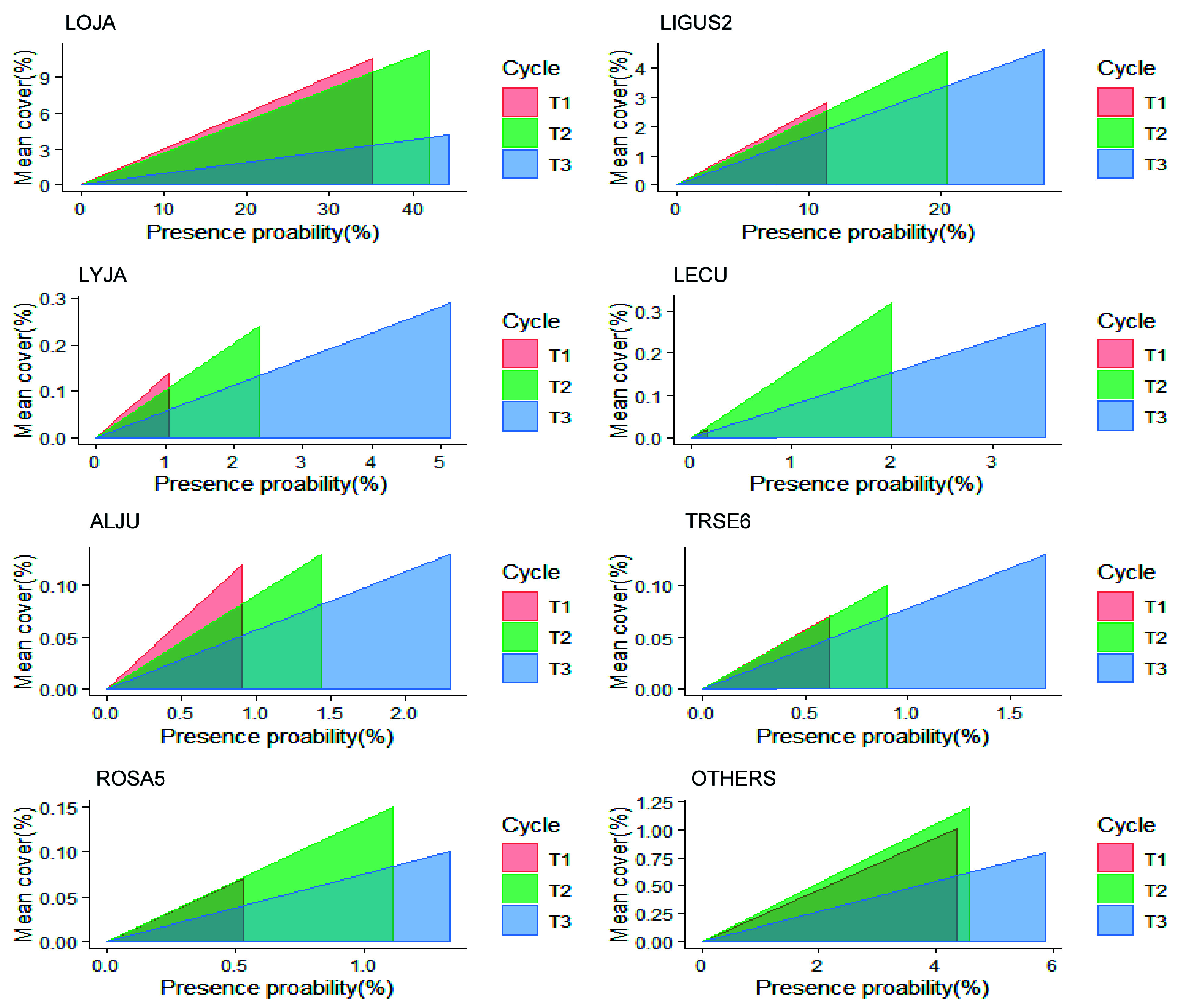
Presence probability (x-axis) and mean cover percent (y-axis) of major NNIPS across all FIA subplots in Alabama's forestlands. NNIPS names in this figure based on FIA Vegetation Species Code (VEG SPCD); LOJA (Japanese honeysuckle), LIGUS2 (Privet), LYJA (Japanese climbing fern), LECU (Chinese lespedeza), ALJU (Silk-tree), TRSE6 (Chinese tallow tree), ROSA5 (Rose), and OTHERS (all other nonnative invasive plant species). The area inside triangles represents the invasion index (severity) and the shape of triangles represents whether an NNIP species is a fast-spreading species (larger changes in the presence probability) or a fast-establishing species (larger changes in the cover percentage) between different inventory cycles.

The number of infested subplots in Alabama in each cycle is given in Table 3. The abundance of all major NNIPS increased over time. Japanese honeysuckle was the most abundant species, followed by privet and Japanese climbing fern. The change in Japanese honeysuckle abundance over time was nearly 19% between T1 and T2 and 5% between T2 and T3. The second most abundant species, privet, increased by almost 80% between T1 and T2 and 36% between T2 and T3. The rate of increment on less abundant species increased at a higher rate. In general, the average cover percent of these species increased over time. However, the average cover percent of Japanese honeysuckle decreased between T2 and T3 (Fig. 3 & Table 3).

**Table 3:** Number of infested subplots by major NNIPS in Alabama's forestlands.

FIA species code	Common name	Latin name	Form	Infested subplot count				Presence probability (%)				Mean cover (%)		
				T1	T2	T3		T1	T2	T3		T1	T2	T3
LOJA	Japanese honeysuckle	*Lonicera japonica* Thunb	Vine	5,348	6,400	6,751		35.09	41.99	44.30		10.58	11.18	4.20
LIGUS2	Privet	*Ligustrum* L.	Shrub	1,740	3,122	4,248		11.42	20.49	27.87		2.80	4.55	4.59
LYJA	Japanese climbing fern	*Lygodium japonicum* (Thunb.)	Fern	162	360	781		1.06	2.36	5.12		0.14	0.24	0.29
LECU	Chinese lespedeza	*Lespedeza cuneata* (Dum. Cours.)	Forb	23	303	537		0.15	1.99	3.52		0.02	0.32	0.27
ALJU	Silk-tree	*Albizia julibrissin* Durazz.	Tree	139	219	351		0.91	1.44	2.30		0.12	0.13	0.13
TRSE6	Chinese tallow tree	*Triadica sebifera* (L.) Small	Tree	95	137	255		0.62	0.90	1.67		0.07	0.10	0.13
ROSA5	Rose	*Rosa* L.	Shrub	81	169	203		0.53	1.11	1.33		0.07	0.15	0.10
Others				663	695	894		4.35	4.56	5.87		1.01	1.21	0.80

### Spatial and temporal patterns of major NNIPS in Alabama

**Figure 4:**
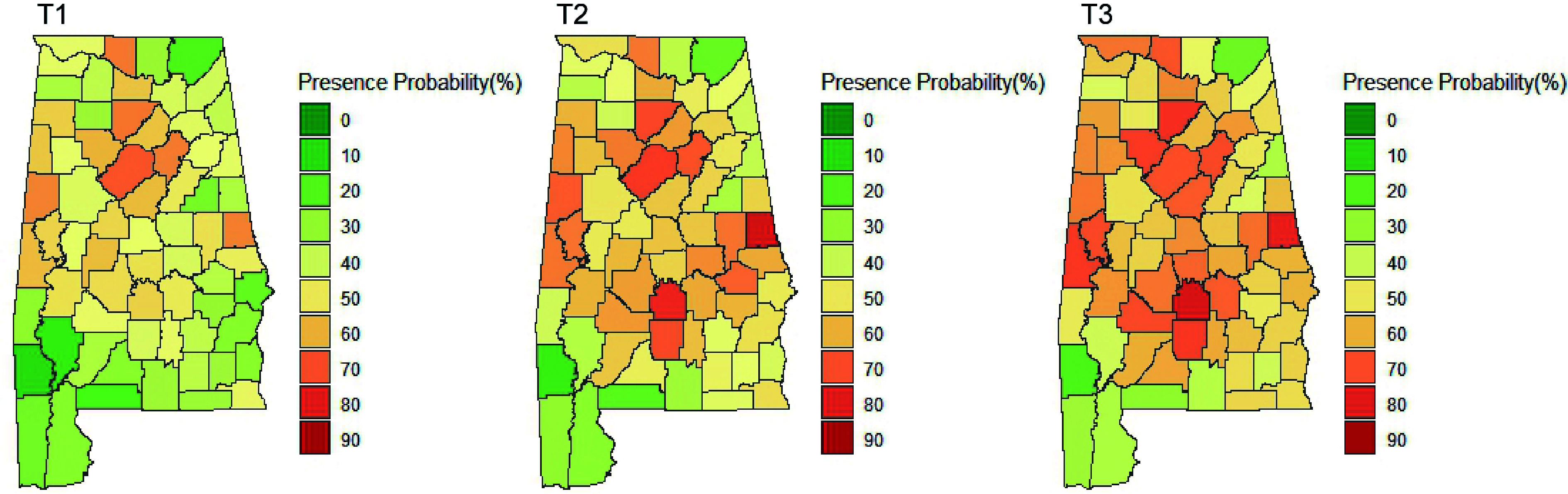
Presence probability of all NNIPS in Alabama's forestlands over time (T1: 2001−2005, T2: 2006−2012, T3: 2013−2019).

**Figure 5:**
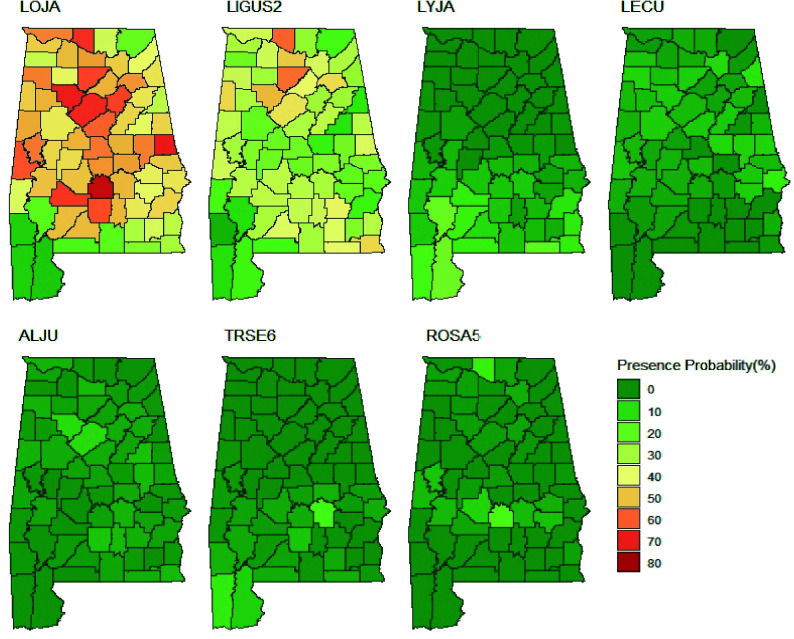
Presence probability (%) of individual NNIPS measured between 2013 and 2019 in Alabama's forestlands. LOJA (Japanese honeysuckle), LIGUS2 (Privet), LYJA (Japanese climbing fern), LECU (Chinese lespedeza), ALJU (Silk-tree), TRSE6 (Chinese tallow tree), and ROSA5 (Rose).

The spatial and temporal visualization of the presence probability of major NNIPS in Alabama is shown in Figs 4 & 5. Figure 4 represents the aggregated presence probability of all species over time across space. The presence probability of individual species can be seen in Fig. 5, which shows the presence probability of individual species based on T3 (i.e., 2013−2019) records.

### Best modeling unit

Among modeling units, Moran's I statistics varied over time. County-level modeling units gave the highest Moran's I statistics for T1 and T3, and the ecological unit-section gave the highest for T2. All hydrological units had a lower Moran's I than county-level modeling units (Table 4). Thus, we chose county-level modeling units for this analysis.

**Table 4:** Moran's I test of the invasion index of all NNIPS in Alabama's forestlands.

Modeling unit	T1				T2				T3		
	Moran's I	Std error	*p*-value		Moran's I	Std error	*p*-value		Moran's I	Std error	*p*-value
HUC 4	−0.24	−0.32	0.63		0.02	0.09	0.18		−0.18	−0.08	0.53
HUC 6	0.14	1.36	0.09		0.32	2.38	0.01		0.18	1.64	0.05
HUC 8	0.27	3.19	< 0.001		0.35	3.98	< 0.001		0.23	2.68	< 0.001
HUC 10	0.36	10.49	< 0.001		0.36	10.36	< 0.001		0.26	7.35	< 0.001
HUC 12	0.24	15.08	< 0.001		0.18	11.59	< 0.001		0.18	11.29	< 0.001
COUNTY	0.40	5.55	< 0.001		0.38	2.25	< 0.001		0.37	5.23	< 0.001
Ecoregion (section)	0.37	2.14	0.02		0.51	2.44	0.01		0.27	2.00	0.02
Ecoregion (subsection)	0.06	0.82	0.21		0.01	0.37	0.35		0.01	0.28	0.39

### Factors influencing NNIPS spread in Alabama

**Figure 6:**
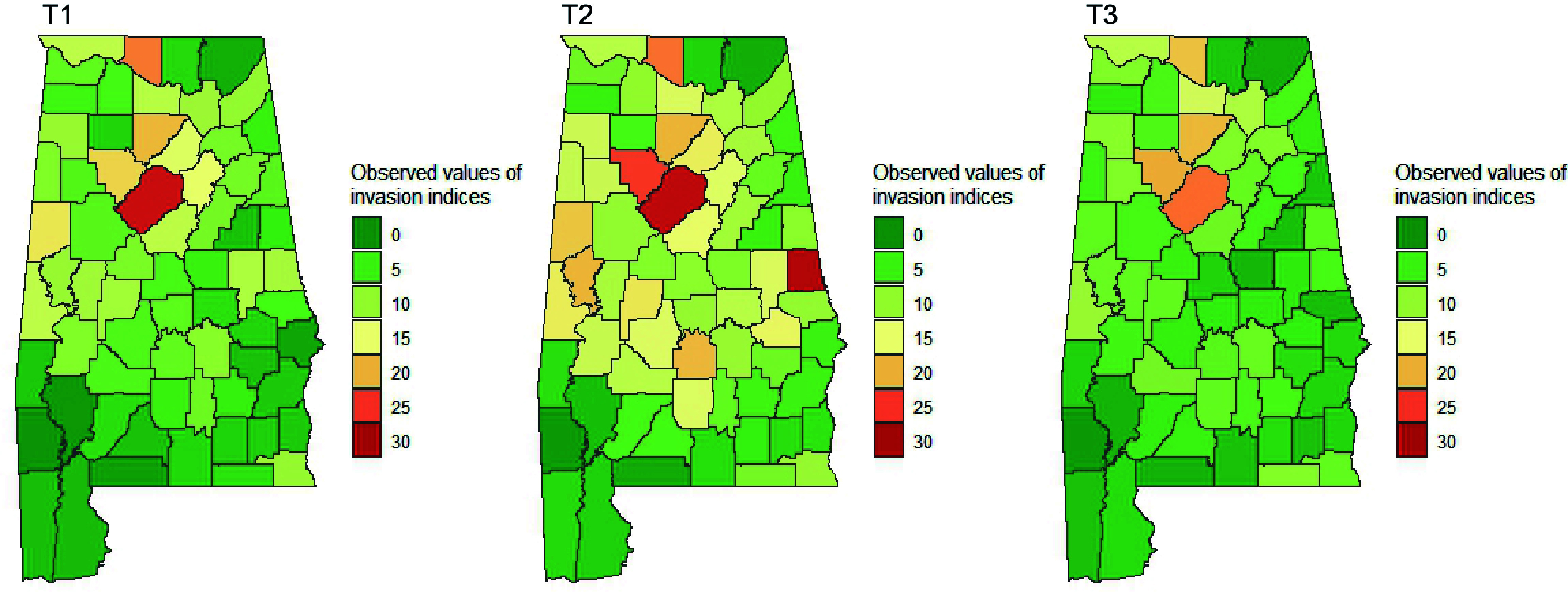
Observed indices of invasion severity over time in Alabama's forestlands. Dark green represents the lowest and red represents the highest level of invasion.

**Figure 7:**
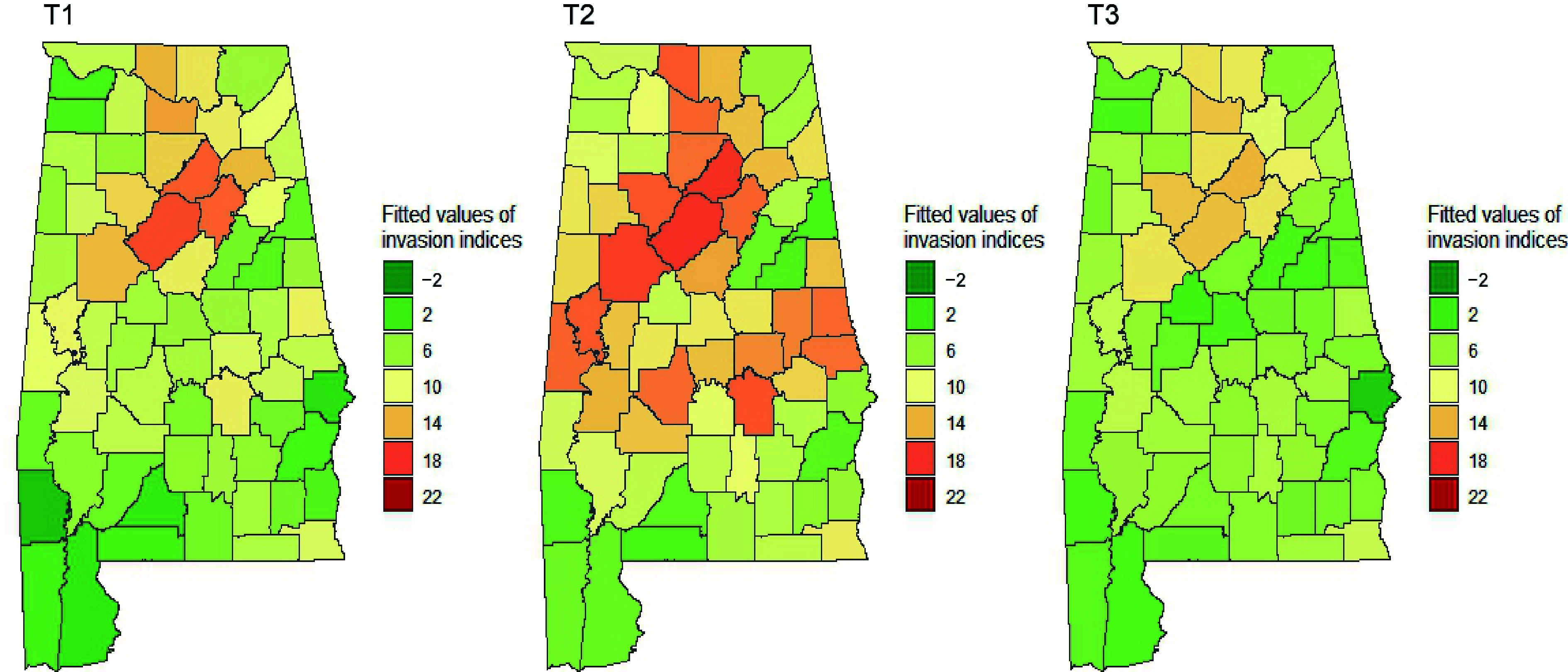
Estimated indices of invasion severity by spatial lag models over time in Alabama's forestlands. Dark green represents the lowest and red represents the highest level of invasion.

**Figure 8:**
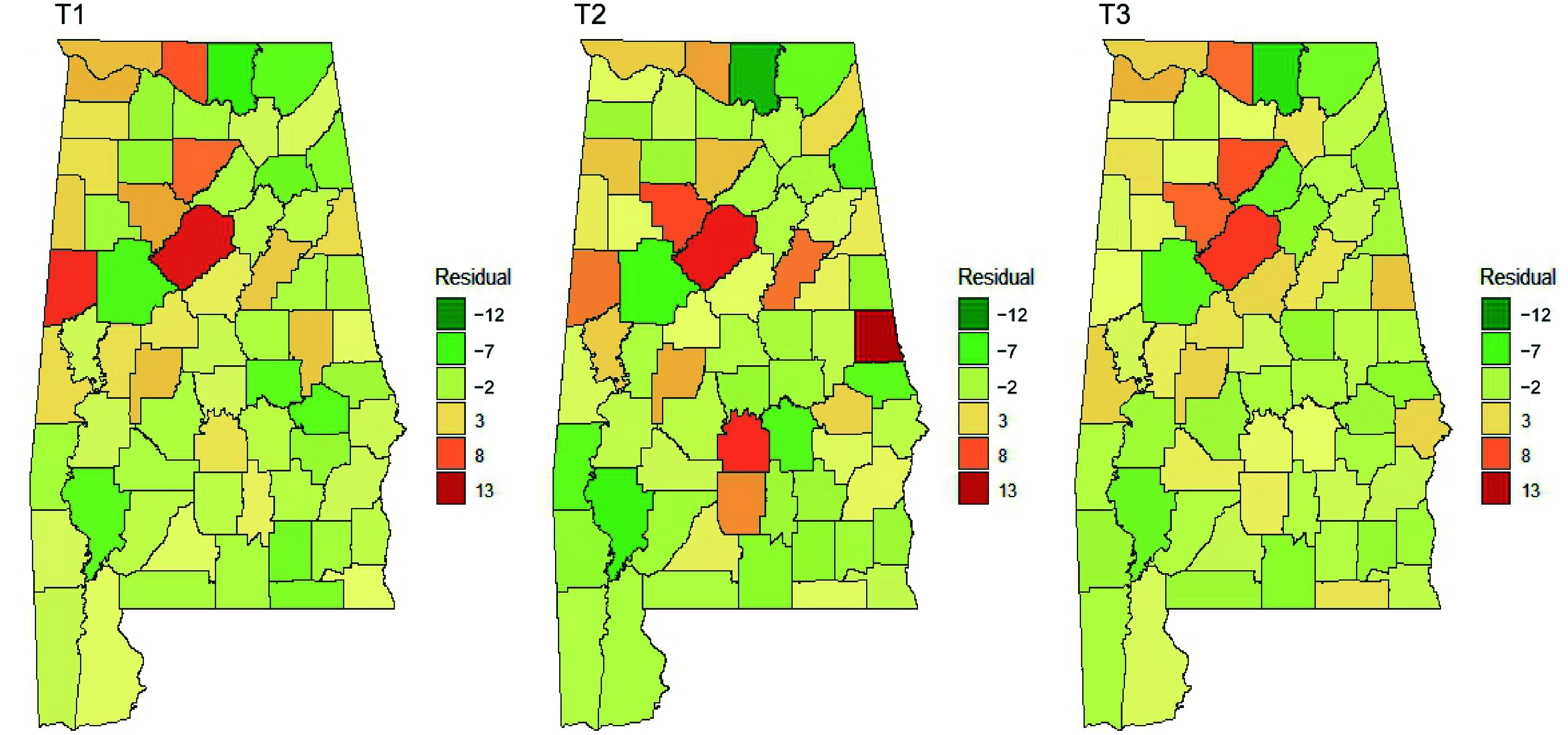
Residuals of spatial lag models over time. The autocorrelation test of residuals is not statistically significant at the significance level of 0.05 (Table 5), which means the spatial lag models well fit the invasion patterns of NNIPS.

We fitted observed invasion indices using the spatial lag model that utilized various independent variables (Table 5). Best-fitted spatial lag models were identified for each measurement based on the lowest AIC. Table 5 shows selected variables and their coefficients, AIC, lag coefficient, and test values of residual autocorrelation. All models had positive and significant lag coefficients. Observed invasion indices, model-predicted values, and residuals of the fitted models are graphically illustrated in Figs 6, 7 & 8.

**Table 5:** Estimated regression coefficients and summary statistics of the fitted spatial lag models.

Measurement cycle (year)	Model statistics	Variable	Estimated coefficients	*p*-value	Residual autocorrelation	
					Test z-value	*p*-value
T1 (2001−2005)	lag coefficient ρ = 0.51 (*p* < 0.001) AIC = 390.5	intercept	4.681	0.085	0.551	0.458
		pop_2010_nbh	0.029	0.020		
		rd_density	19.016	0.041		
		oak_pine	−0.187	0.124		
		lob_hard	−0.273	0.116		
		mix_hard	−0.106	0.139		
		wo_ro_hi	−0.172	0.027		
		public_own_pct	−0.086	0.110		
		ot_pct	−113.519	0.011		
		wa_pct	−21.017	0.085		
T2 (2006−2012)	lag coefficient ρ = 0.53 (*p* < 0.001) AIC = 407.37	intercept	10.264	< 0.001	0.904	0.341
		pop_2010_nbh	0.024	0.058		
		oak_pine	−0.281	0.036		
		wo_ro_hi	−0.209	0.019		
		elm_ash_cot	1.008	0.090		
		public_own_pct	−0.175	0.006		
		ot_pct	−168.324	0.001		
T3 (2013−2019)	lag coefficient ρ = 0.55 (*p* < 0.001) AIC = 367.95	intercept	6.171	< 0.001	0.228	0.633
		pop_2010_nbh	0.029	0.003		
		lob_hard	−0.483	< 0.001		
		wo_ro_hi	−0.161	0.018		
		public_own_pct	−0.122	0.007		
		ot_pct	−80.554	0.034		
		wa_pct	−14.603	0.127		

Demography (pop_2010_nbh) and ownership were the only variables shown in all spatial lag models, suggesting their roles in NNIP invasions were universal and perpetual over time. NNIP invasion index (severity) increased with the county population. NNIPs are less likely to invade public forestlands than private lands. Forest types (groups) also played a significant role, but NNIP invasion severity by forest type (group) varied over time (Table 5).

## Discussion

In Alabama, NNIP species are spreading both spatially and increasing in number temporally. This study examined the distribution of major NNIP species, quantified the invasion indices, selected the best modeling units, and developed a model considering the spatial lag effect of invasion indices. We aggregated all major NNIP species and quantified invasion indices using presence probability and cover percent. Hussain et al.^[5]^ used invasive species count data to explore the ecological and economic aspects of invasive species in Alabama. We believe the invasion index used in this study is more meaningful in exploring the invasion severity because it considers two dimensions of the invasion: presence probability (abundance) and cover percent (dominance). Nepal et al.^[35]^ used a similar quantification approach to quantify the invasion severity of Chinese tallow trees.

Our analysis showed that Japanese honeysuckle had been the most prevalent invasive species in Alabama. Miller et al.^[36]^ also described this species as the most frequent and dense, especially in eastern-central Alabama. Its presence probability was higher than all others' combined values in all measurement periods (Table 4). Similarly, its cover percentage was higher than all others' combined values in T1 and T2. Thus, it has contributed significantly to the overall invasion index in T1 and T2. As we noted, the cover percent of Japanese honeysuckle was meager in T3 compared to other measurement cycles (Fig. 3); thus, the overall invasion index was lower in T3. Japanese honeysuckle is likely to be deciduous in response to drought or cold, even though it is an evergreen or semi-evergreen species^[37]^. It is uncertain if the abrupt drop in cover percent observed in T3 was due to this species' deciduous nature or any other reasons such as the competition with a developing forest overstory. Future research should focus on why Japanese Honeysuckle's cover percentage was lower in T3. Japanese honeysuckle is normally constant across the landscape, with little increase or decrease; the giant swing in the data is most likely attributable to the change in field guide protocols (Personal communication with Alabama Forestry Commission).

The number of NNIPS has been increasing over time in Alabama. We found 25 NNIPS in T1, 26 in T2, and 33 in T3. The current non-completed cycle (2020 and 2021; i.e., T4) - has already reported 26 distinct species. In T3 alone, FIA data showed seven new species that were not present during the T2 measurement. The increase during the third measurement indicates that nonnative species are spreading across Alabama. As new invasive species establish themselves in Alabama, existing invasive species continue to spread. It leads to widespread invasion presence but at a relatively lower rate of increase in cover percentage.

Choosing appropriate modeling units is vital because spatial aggregation impacts spatial autocorrelation and estimated coefficients^[38]^. Using Moran's I value as our guide, we selected the county-level modeling unit. County-level modeling units perform better than other modeling units for aggregated invasion indices of all NNIPS. However, modeling units might be different for the invasion index of individual NNIPS. For example, the Chinese tallow invasion index performed better with the hydrological unit HUC8. It may be related to dispersal factors associated with Chinese tallow. Birds and water currents mainly influence its dispersal following flooding^[39, 40]^.

Hussain et al.^[5]^ found that forest ownership and proximity to densely populated areas in Alabama positively impacted the occurrence and abundance of invasive species. They modeled occurrence and abundance separately based on zero-inflated negative binomial regression. To account for the neighboring effect, we developed an invasion index accounting for both occurrence and abundance in the spatial lag model. Thus, our model can better explain the influencing factors of invasions in Alabama. Further, we ran the model across three different measurement data sets. Our results show that all three spatial lag models (Table 5) had a positive and significant lag coefficient (ρ). These indicate positive spatial feedback. A higher invasion index in a county also raises the neighboring counties' predicated invasion index.

We found that publicly owned forest cover percentage negatively impacted invasion indices across measurement periods. Zhai et al.^[6]^ & Hussain et al.^[5]^ found similar outcomes in the southern states, with areas under private ownership more likely to have more NNIPS. We also found that the neighbor county population density positively impacted the invasion index. The positive relation between the invasion index and population is likely due to infrastructures facilitating the dispersal of NNIPS^[41]^. Furthermore, Chen also observed a positive relationship between invasive species richness and total road length in Alabama^[42]^. The amount and type of forest cover also impact the invasion index. For instance, the increasing cover percent of white oak/red oak/hickory forests was likely to decrease the invasion index in Alabama (Table 5).

## Conclusions

This study obtained NNIPS data from more than 5,000 remeasured FIA plots across Alabama. We mapped major NNIPS in both spatial and temporal domains. We observed that NNIPS spread across the state and that invasion severity increased over time. Japanese honeysuckle was the most widespread species across the state. We quantified the invasion index/severity based on cover percent and presence probability. Invasion indices were quantified individually for all multiple modeling units: five levels of hydrological units, three levels of ecological units, and county. Moran's I test showed that the county-level modeling units had the highest spatial autocorrelation; thus, we chose the county-level modeling unit best suited for the spatial lag model. The spatial lag model suggests that forested area, forest ownership, and neighbor's population density significantly impact the invasion severity in Alabama. We suggest invasive species controlling practices should focus on both within the county and surrounding counties.

## Author contributions

The authors confirm contribution to the paper as follows: study design, discussion, and revision: Fan Z; data analysis and writing: Nepal S; revision and discussion: Spetich MA. All authors reviewed the results and approved the final version of the manuscript.

## Data availability

The USDA Forest Service's Forest Inventory and Analysis (FIA) datasets of nonnative invasive plant species used for this study are available to the public from the website: www.fs.usda.gov/research/products/dataandtools/datasets/fia-datamart.
